# Neurofibromin Loss of Function Drives Excessive Grooming in *Drosophila*

**DOI:** 10.1534/g3.115.026484

**Published:** 2016-02-18

**Authors:** Lanikea B. King, Marta Koch, Keith R. Murphy, Yoheilly Velazquez, William W. Ja, Seth M. Tomchik

**Affiliations:** * Department of Neuroscience, The Scripps Research Institute, Scripps Florida, Jupiter, Florida 33458; ‡ Department of Metabolism and Aging, The Scripps Research Institute, Scripps Florida, Jupiter, Florida 33458; ** SURF Program, The Scripps Research Institute, Scripps Florida, Jupiter, Florida 33458; † Laboratory of Glia Biology, Center for the Biology of Disease, VIB, 3000 Leuven, Belgium; § Integrative Biology Graduate Program, Florida Atlantic University, Jupiter, Florida 33458

**Keywords:** *Drosophila*, grooming, neurofibromatosis, Nf1

## Abstract

Neurofibromatosis I is a common genetic disorder that results in tumor formation, and predisposes individuals to a range of cognitive/behavioral symptoms, including deficits in attention, visuospatial skills, learning, language development, and sleep, and autism spectrum disorder-like traits. The *nf1*-encoded neurofibromin protein (Nf1) exhibits high conservation, from the common fruit fly, *Drosophila melanogaster*, to humans. *Drosophila* provides a powerful platform to investigate the signaling cascades upstream and downstream of Nf1, and the fly model exhibits similar behavioral phenotypes to mammalian models. In order to understand how loss of Nf1 affects motor behavior in flies, we combined traditional activity monitoring with video analysis of grooming behavior. In *nf1* mutants, spontaneous grooming was increased up to 7x. This increase in activity was distinct from previously described dopamine-dependent hyperactivity, as dopamine transporter mutants exhibited slightly decreased grooming. Finally, we found that relative grooming frequencies can be compared in standard activity monitors that measure infrared beam breaks, enabling the use of activity monitors as an automated method to screen for grooming phenotypes. Overall, these data suggest that loss of *nf1* produces excessive activity that is manifested as increased grooming, providing a platform to dissect the molecular genetics of neurofibromin signaling across neuronal circuits.

Multiple human genetic disorders result in cognitive dysfunction. One of the most common inherited genetic disorders is neurofibromatosis, type I (NF-1), a disorder characterized by nerve sheath tumors, and other visible characteristics affecting the skin and eyes. In addition, some form of cognitive dysfunction is present in approximately 80% of individuals with NF-1, making it the most common monogenic disorder that affects cognitive function (Hyman *et al.* 2005; Diggs-Andrews and Gutmann 2013). Cognitive symptoms vary, but can include deficits in general intellectual functioning, visual perception, language, executive function, attention, cognitive flexibility, learning, and sleep patterns, as well as features of autism spectrum disorder (Hyman *et al.* 2005, 2006; Garg *et al.* 2013; Walsh *et al.* 2013). Due to their effect on quality of life, these complications are considered among the highest causes of lifetime morbidity in individuals with NF-1 (Ozonoff 1999; Hyman *et al.* 2005; Payne 2013). Consequently, much research has focused on the neurobiological basis of NF-1 cognitive phenotypes (Hofman *et al.* 1994).

Animal models recapitulate some behavioral features of the NF-1 phenotypic spectrum. Mice and flies with mutations in the *nf1* gene exhibit reduced performance in learning and memory assays (Guo *et al.* 2000; Costa *et al.* 2001; Ho *et al.* 2007; Buchanan and Davis 2010; Gouzi *et al.* 2011). In addition, *nf1* mutant flies exhibit defects in growth (The *et al.* 1997; Gouzi *et al.* 2011; Walker *et al.* 2013), circadian rhythms (Williams *et al.* 2001), and activity across the day/night cycle (Williams *et al.* 2001; van der Voet *et al.* 2015). Nf1 is a Ras-GAP, functioning as a critical inhibitor of Ras-GTPase signaling (Cichowski and Jacks 2001; Costa *et al.* 2002). Consequently, treatment of Nf1-deficient animals with statins to prevent hyperactivation of Ras can ameliorate some of the aberrant behavioral phenotypes in animal models (Li *et al.* 2005). However, clinical trials using lovastatin or simvastatin to treat NF-1 in humans have seen mixed success thus far (Krab *et al.* 2008; Acosta *et al.* 2011; Chabernaud *et al.* 2012; Mainberger *et al.* 2013; van der Vaart *et al.* 2013), leaving patients with no current treatment for the cognitive complications of Nf1 NF-1. In addition, Nf1 deficiency decreases cAMP levels, possibly indirectly (The *et al.* 1997; Tong *et al.* 2002; Dasgupta *et al.* 2003; Hannan *et al.* 2006; Brown *et al.* 2010; Diggs-Andrews *et al.* 2013; Walker *et al.* 2013). The complexity of the signaling cascades implicated in NF-1 pathophysiology—Ras, cAMP, and multiple downstream cascades—combined with the lack of drugs to target Nf1 directly, highlights the pressing need for new screening approaches to target NF-1 phenotypes.

To develop a platform that allows rapid, semi-automated screening for modifiers of neuronal dysfunction in NF-1, we examined *Drosophila* locomotor activity, grooming, and sleep by pairing traditional activity monitoring with video tracking and analysis. Studies in *Drosophila* have been instrumental in characterizing the signaling downstream of Nf1 (McClatchey 2007), and development of rapidly quantifiable behavioral phenotypes will likely allow further elucidation of the signaling disruptions underlying the disorder. Here, we report that mutation or knockdown of Nf1 produces a robust increase in spontaneous grooming behavior, in addition to decreased sleep and locomotor activity across both day and night periods. Thus, the *Drosophila* model of NF-1 exhibits phenotypic similarity to particular behaviors associated with frequently comorbid conditions in humans, including autism spectrum disorder (repetitive stereotyped behavior), and attention deficit hyperactivity disorder (ADHD) (increased activity, manifested largely as grooming). These phenotypes provide a platform to dissect the alterations in signaling cascades, and ultimately neuronal function, that result from neurodevelopmental disorders.

## Materials and Methods

### Fly strains

Flies were raised on cornmeal/agar food medium according to standard protocols. They were housed in incubators (Darwin Chambers) maintained at 25°, 60% relative humidity, and kept on a 12:12 light:dark cycle. The *nf1*^P1^ mutation was backcrossed for six generations into the *wCS10* genetic background. Dopamine transporter, fumin (*fmn*), mutant flies were compared to their control strain *w*^1118^ (Kume *et al.* 2005). An Nf1 RNAi line was obtained from the Vienna Drosophila RNAi Center (VDRC #109637), and uas-dicer2 was used in all crosses to enhance the RNAi effect (Dietzl *et al.* 2007). The empty attP control line (VDRC #60100) was used in gal4/+ control crosses to ensure a matched genetic background across all groups. Male flies were used for all experiments to prevent egg accumulation in the activity monitors.

### Activity monitoring

Infrared beam crossing was monitored with *Drosophila* activity monitors (Trikinetics). The DAM2 (upright) model was used in experiments not requiring video monitoring. Glass tubes were prepared as follows. Each tube was punched through a 1 cm thick piece of room-temperature (hardened) food containing 5% sucrose and 2% agar. This food plug was covered with a black cap to reduce desiccation, and an EPDM rubber O ring was fitted to the outside of the tube to maintain lateral alignment in the monitor. Flies were anesthetized with CO_2_, and males were collected into DAM tubes, with one genotype loaded per monitor. Each monitor was placed into the incubator perpendicular to, and equidistant from, the white LED light source in the incubator. Activity was recorded with a 1 min sampling interval using the native Trikinetics software, and combined into standard 30 min bins for plotting in some figures as noted. Data from the first (partial) day/night period was excluded from analysis. Data were collected for 5 d. Day and night activity counts were summed for each fly, independent of day. For each fly, data from each time bin was averaged across 5 d, and then these data points were averaged across flies, to plot day and night activity across zeitgeber time. Custom Matlab (Mathworks) scripts were used to plot sleep and activity profiles. Sleep was calculated using a standard 5 min inactivity window (Shaw *et al.* 2000).

### Combined activity monitoring/video recording

To monitor the activity and grooming of flies in activity monitors simultaneously, the DAM4 (flat) model was used. Four male flies were loaded into glass DAM tubes (as above, except that the O ring was omitted), and placed into a DAM4 monitor. Beam crossings were recorded in 10 s bins using the native Trikinetics software. The black area below each tube in the monitor was covered with white tape to enhance the contrast between the fly and background. The incubator was illuminated during the 12 hr day cycle with white LEDs (380 lux at the monitor location), with separately controlled red LEDs providing 24 hr illumination for video monitoring (combined red/white: 485 lux). Two monochrome Firefly MV 1394a cameras (Point Gray) were used to collect videos of the flies in the tubes, in 10 min increments, for 24 hr in total. The cameras were mounted in the incubator, 6 cm from the top of the DAM4 monitor. Each camera was fitted with a Fujinon YV2.8 × 2.8SA-2 lens, and focused on the center of the tubes. Videos were collected with a custom Matlab (Mathworks) script, using the Image Acquisition Toolbox, at 7.5 frames per sec with Motion JPEG 2000 compression. Four flies were captured per experiment (two per camera). Videos were observed offline and compared to the activity trace recorded by the monitor. All monitor-recorded events were scored as either grooming (fly stationary in/near IR beam, visibly cleaning head, legs, wings, or thorax/abdomen), or locomotion (walking through IR beam) by an observer blind to the genotype.

### Open-field grooming video capture

An open field arena was constructed, 2.85 mm in height and 25.4 mm in diameter, consisting of an opaque (white) acrylic lateral boundary covered on the top and bottom with two clear polycarbonate sheets. The apparatus was illuminated from below with white LEDs that were filtered through a sheet of white acrylic; the light intensity was measured at 720 lux in the location of the fly. A 1394a camera (as above) was mounted 5 cm above the arena. A single male fly was loaded into the arena with an aspirator, and recorded at 7.5 frames per second, 640 × 480 with Motion JPEG 2000 compression. Two videos were collected for each fly, one immediately after loading into the chamber (0–5 min), and one after a 15 min acclimation period (15–20 min). Videos of control and experimental genotype flies were alternated to distribute any circadian variation equally across all groups. Manual scoring of videos was carried out by an observer blind to the genotype. Start and stop frames were noted for each grooming event, which was further categorized according to which body part the fly was grooming: front legs, head/eye, abdomen, wings, or hind legs (Seeds *et al.* 2014). Total grooming time was calculated as the sum of all grooming events.

### Statistical analysis

Statistical analyses were carried out in Graphpad Prism. Data were considered normally distributed if no significant deviation from normality was detected by the D’Agostino-Pearson omnibus test. Normally distributed data were compared with Student’s *t*-test or ANOVA followed by Tukey *post hoc* tests. Data that deviate from normality were tested with the Kruskal-Wallis test followed by Dunn’s post tests. Two-way analyses were carried out with a two-way repeated-measures ANOVA followed by Sidak’s multiple comparison tests. Results with RNAi experiments were considered positive only if the RNAi experimental group differed significantly from both the gal4/+ and uas/+ controls. Exponential curves were fit to mean values of grooming percentage histograms following the equation: *y* = C(1 – r *e*^–k^*^t^*), where the upper bound C = 100, *t* = time, and r and k are constants. Mean frequency (Figure 2, E and I, and Figure 4E) was calculated as the average of all nonzero activity bins from each fly (*n* = 30–32 per genotype). Fly strains and Matlab scripts are available upon request.

### Data availability

The authors state that all data necessary for confirming the conclusions presented in the article are represented fully within the article.

## Results

### Reduction of neurofibromin alters activity and reduces sleep across the diurnal photoperiod

**Figure 1:**
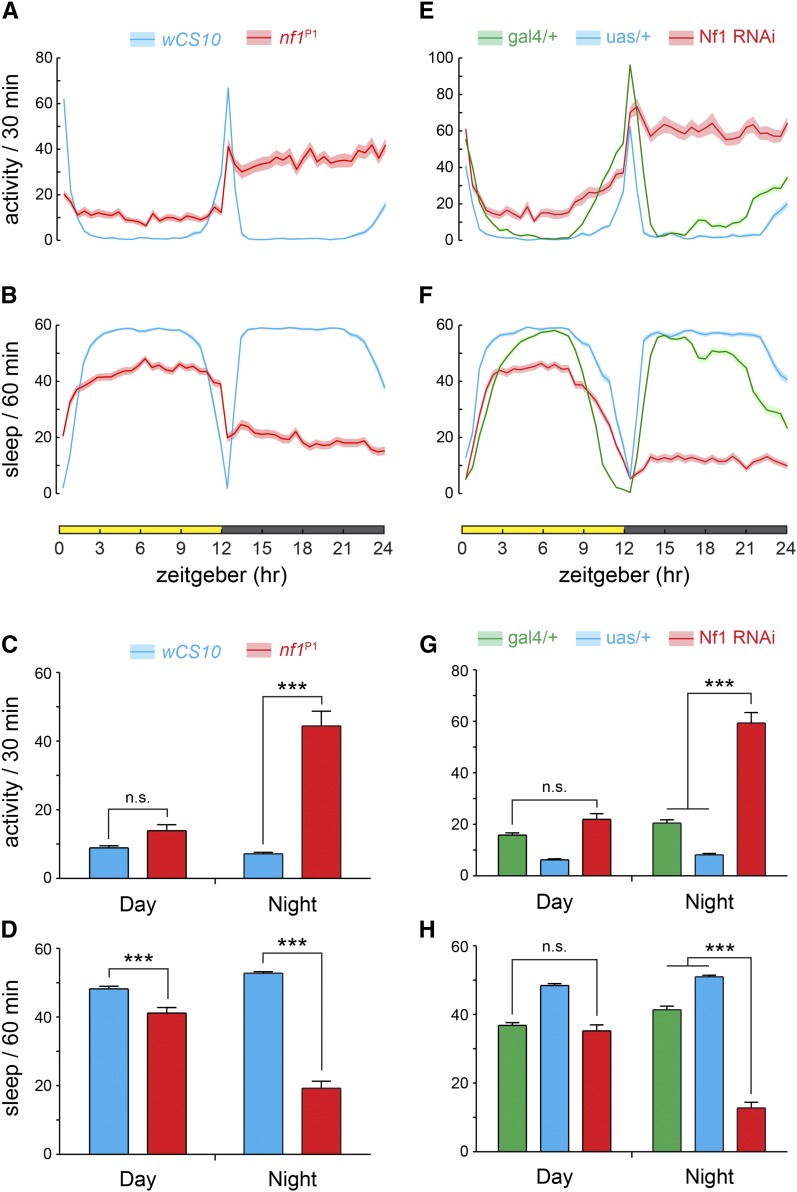
Nf1 mutants and RNAi lines exhibit increased activity and loss of sleep mainly at night, as measured by infrared activity monitors. (A) Activity of *nf1*^P1^ (*n* = 31) and control *wCS10* (*n* = 32) flies across the diurnal cycle, in units of beam counts per 30 min bin. Lines and shading represent mean ± SEM. (B) Sleep plots of the *nf1*^P1^ mutants and *wCS10* controls. (C) Daytime and nighttime activity for *nf1*^P1^ mutants and *wCS10* controls. Bars and whiskers represent mean ± SEM. *** *P* < 0.001 (ANOVA/Sidak); n.s., not significant. (D) Daytime and nighttime sleep for *nf1*^P1^ mutants and *wCS10* controls. *** *P* < 0.001 (ANOVA/Sidak). (E) Activity in Nf1 RNAi lines and controls across the diurnal cycle, in units of beam counts per 30 min bin. Full genotypes: gal4/+ = w;uas-dcr2/+;nSyb-gal4/+ (*n* = 31), uas/+ = w;uas-dcr2/uas-nf1RNAi (*n* = 32), Nf1 RNAi = w;uas-nf1RNAi/uas-dcr2;nSyb-gal4/+ (*n* = 32). (F) Sleep plots of the Nf1 RNAi lines and controls. (G) Daytime and nighttime activity for Nf1 RNAi lines and controls. *** *P* < 0.001 (ANOVA/Sidak); n.s., not significant. (H) Daytime and nighttime sleep for Nf1 RNAi lines and controls. *** *P* < 0.001 (ANOVA/Sidak); n.s., not significant.

NF-1 is highly comorbid with ADHD, suggesting that changes in arousal and activity may be a feature of the disorder. To characterize how neurofibromin affects activity patterns across the diurnal cycle in *Drosophila*, we placed flies into infrared activity monitors that measure the number of times a fly crosses an infrared beam in the center of a glass tube (Figure 1). Beam breaks were recorded, and sleep calculated according to the standard criterion of 5 min of inactivity (Shaw *et al.* 2000). First, we tested whether *nf1*^P1^ mutant flies, which harbor a large deletion in the *nf1* locus, including the catalytic GAP-related domain (The *et al.* 1997), exhibit any difference in activity and/or sleep compared to wild-type controls. The mutants exhibited an increase in beam crossings at night (with a trend during the day), representing an apparent increase in activity relative to the *wCS10* controls (Figure 1, A and C). In addition, there was significant loss of sleep across both day and night periods (Figure 1, B and D). To confirm these results with an independent loss-of-function approach, we knocked down Nf1 with RNAi (VDRC #109637). Similar to a previous report (van der Voet *et al.* 2015), we observed that pan-neuronal knockdown of Nf1 produced an increase in activity and loss of sleep, which were significant only at night (Figure 1, E–H). These data suggest that Nf1 loss of function increases activity and decreases sleep.

### Activity in Nf1 mutants is clustered in bursts that represent grooming behavior

**Figure 2:**
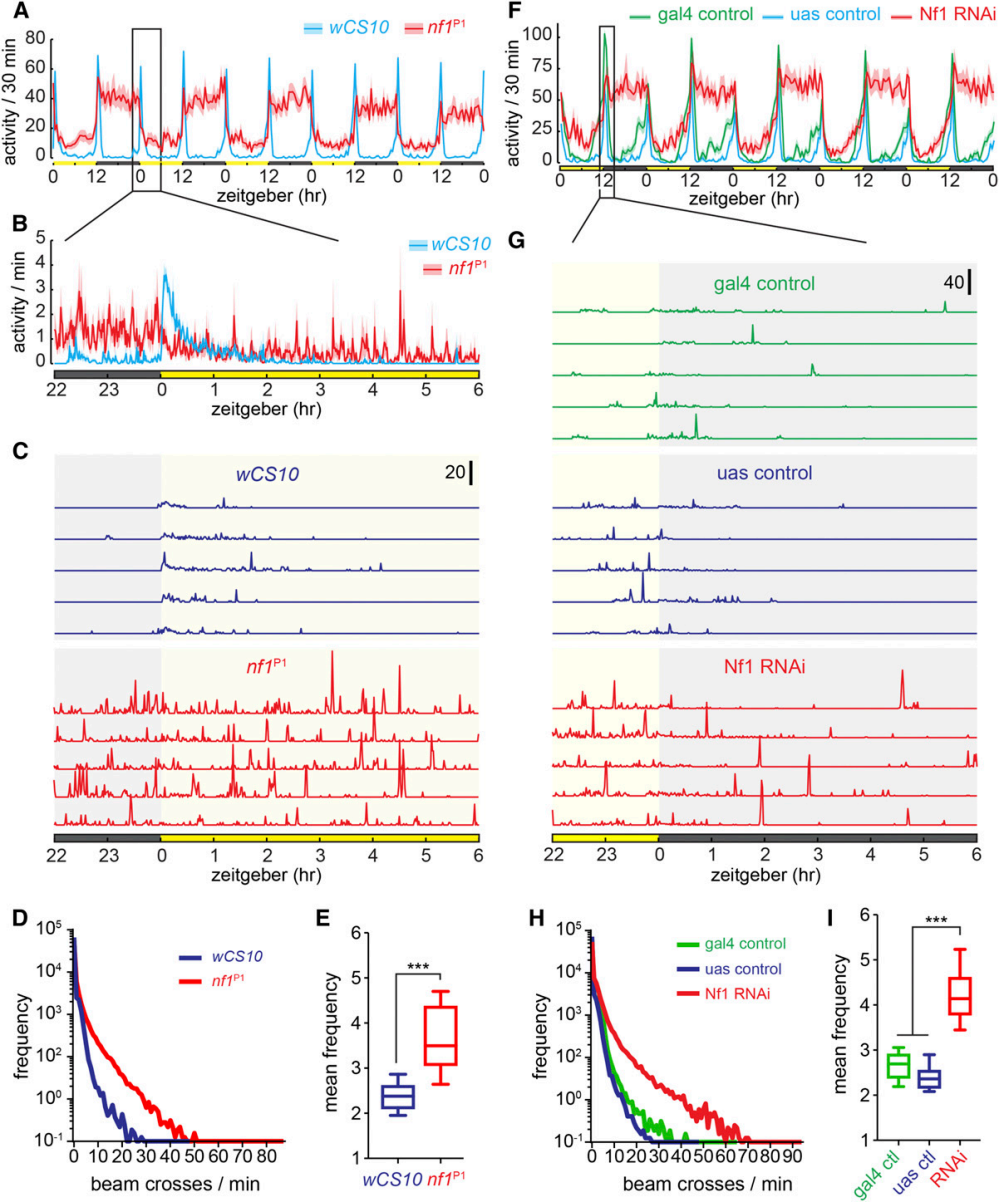
Increased activity with Nf1 loss of function occurs in concentrated bursts of high-frequency activity, as measured by infrared activity monitors. (A) Activity of *nf1*^P1^ mutants and *wCS10*, in units of beam counts per 30 min. Lines and shading represent mean ± SEM. Data are from the same flies as in Figure 1. (B) Expanded view of an 8 hr time segment of the data shown in (A), in units of beam crossings per 1 min. Lines and shading represent mean ± SEM of activity from all flies. (C) Representative examples of activity traces from individual flies sampled from the group data in (B). The *y*-axis scale bar indicates 20 beam crossings per 1 min. (D) Histogram of activity across flies. Note the log scale. (E) Mean frequency (mean of nonzero values recorded by the infrared activity monitor). *** *P* < 0.001 (Student’s *t*-test). (F) Activity of Nf1 RNAi lines and controls, in units of beam counts per 30 min. Data are from the same flies as in Figure 1. (G) Representative examples of activity traces from individual flies sampled from the data in (F). The *y*-axis scale bar indicates 40 beam crossings per 1 min. (H) Histogram of activity across flies. Note the log scale. (I) Mean frequency (mean of nonzero values recorded by the infrared activity monitor). *** *P* < 0.001 (Kruskal-Wallis/Dunn).

Analysis of mean group data from activity monitors has been shown to mask variation between animals, and/or the temporal structure of the activity traces within animal (Lazopulo *et al.* 2015). Therefore, to more completely characterize the Nf1 activity phenotype, we analyzed single fly activity traces at higher temporal resolution (1 min bins), focusing on the time period surrounding the transition from light to dark (when flies are most active). As expected, control flies showed increases in baseline activity around the evening lights-off transition (Figure 2, A, B, and F). In contrast, *nf1* mutants and RNAi lines did not exhibit a clear peak, consistent with their general arrhythmicity (Williams *et al.* 2001). Superimposed on the basal activity were large spikes, which occurred at irregular intervals (Figure 2, B, C, and G). These spikes were larger in magnitude and more frequent in the *nf1*^P1^ mutants and RNAi line than in controls (Figure 1, C and G). Histograms of the *nf1* mutants and RNAi lines showed noticeably more high-frequency beam crossing events than controls (Figure 2, D and H), and the mean frequency was significantly higher (Figure 2, E and I). The increases in activity observed in mean frequency traces were therefore due, at least in part, to the averaging of relatively sparse but large bursts of activity that were more frequent in Nf1 loss-of-function conditions. Some of these activity spikes were very large (Figure 2, C, D, G, and H), suggesting that they did not reflect the fly patrolling back and forth in the tube (which would generate consistent, lower-frequency elevation of baseline activity, similar to the peaks at the D:L and L:D transitions; Figure 2, C and G).

**Figure 3:**
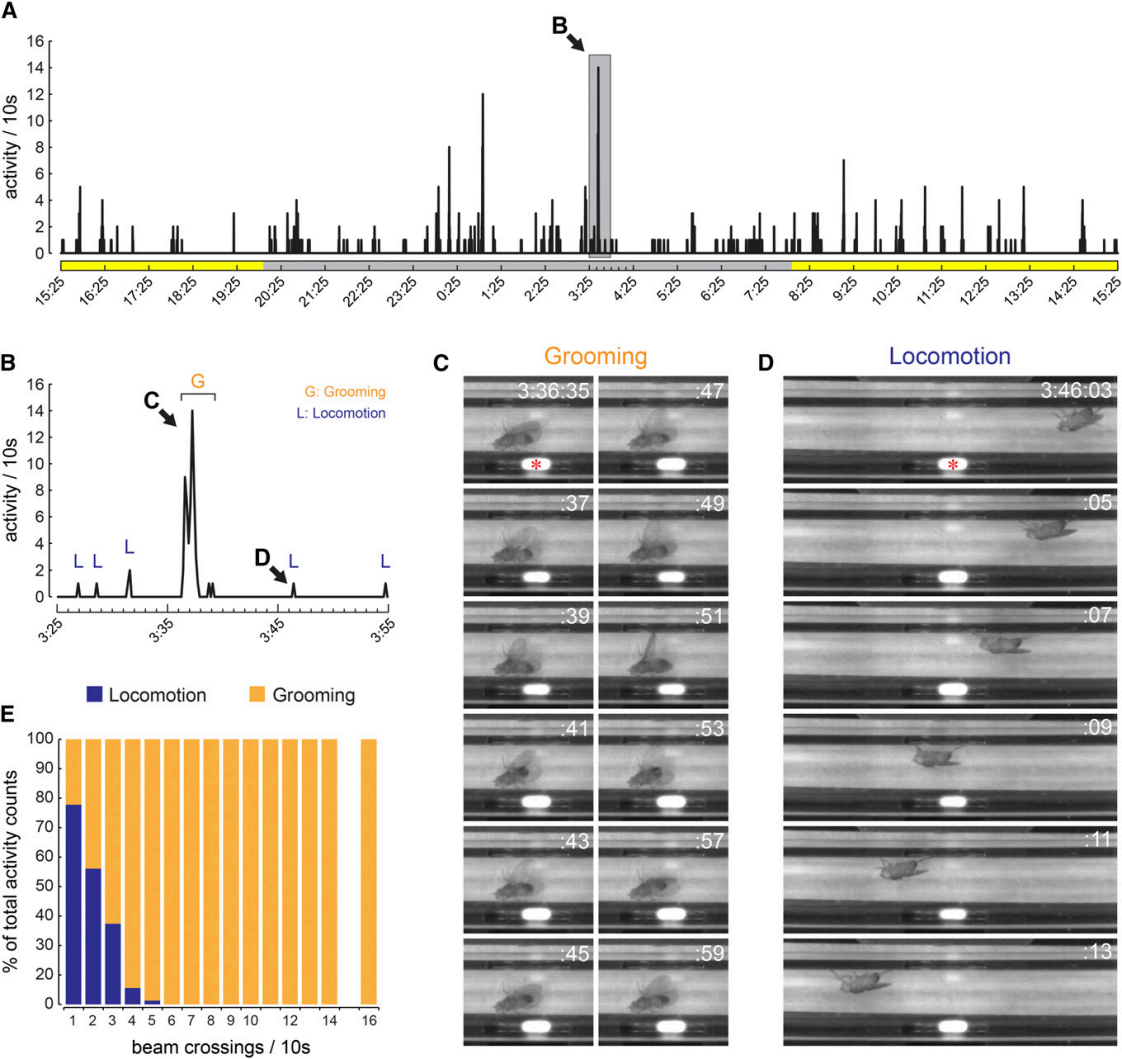
Grooming underlies high-frequency beam crossing events in the infrared activity monitor. (A) Activity of a single *nf1*^P1^ fly over 1 d in the activity monitor, in units of beam counts per 10 s bin. The time region expanded in (B) is highlighted with a gray box. (B) Expanded time scale of the 30 min segment highlighted in (A), spanning three 10 min videos. The peaks highlighting grooming and locomotion in (C) and (D) are indicated with arrows. (C) Video stills of a fly grooming next to the infrared beam. The beam breaks were recorded as a large series of activity peaks by the monitor trace in (A) and (B). The IR beam is marked with an asterisk in the first frame. (D) Video stills of a fly walking past the beam. The beam break was recorded as a single peak in the activity trace, marked in (B). The IR beam is marked with an asterisk in the first frame. (E) Proportion of locomotion and grooming events graphed against beam crossing frequency.

**Figure 4:**
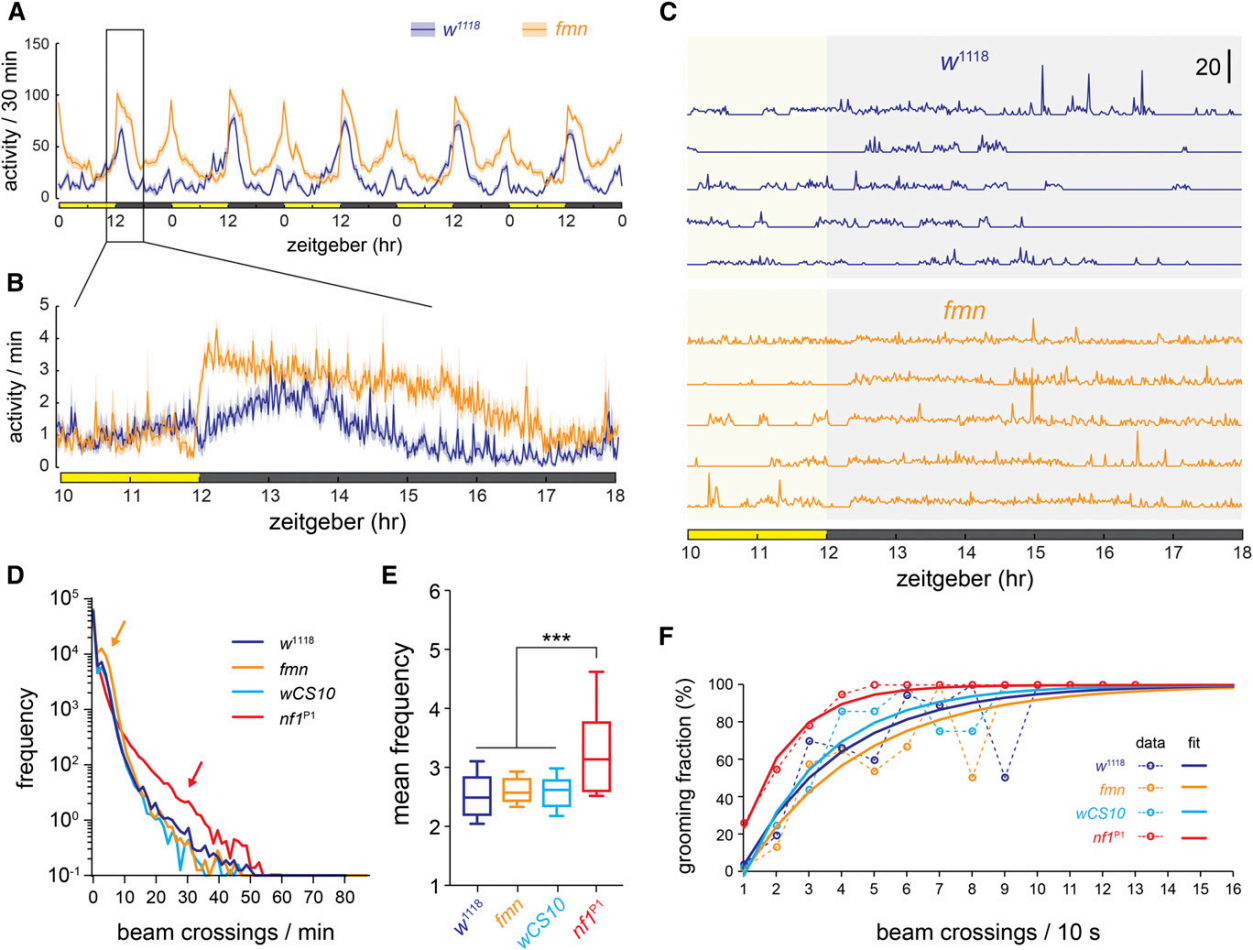
Activity profiles of *nf1*^P1^ mutants differ from the hyperactive *fmn* mutants. *nf1*^P1^ mutants exhibited in increases in high-frequency beam crossing (grooming), while *fmn* mutants exhibited increases in low-frequency beam crossing. (A) Group activity data from *fmn* mutants, in units of beam counts per 30 min bin. Lines and error bars represent mean ± SEM. (B) Group activity data of *fmn* mutants from a 6 hr window in units of beam counts per 1 min bin. (C) Representative examples of activity traces from individual flies sampled from the group data in (B). (D) Histogram of activity of *fmn* and *nf1*^P1^ mutants, with respective wild-type controls. All genotypes were run simultaneously (the *nf1*^P1^ and *wCS10* groups are independent of the data shown in Figure 1 and Figure 2). Note the log scale. Arrows highlight the distinct patterns of *fmn* (orange) and *nf1*^P1^ (red) activity profiles. (E) Mean frequency (mean of nonzero values recorded by the infrared activity monitor). *** *P* < 0.001 (ANOVA/Tukey). (F) Mean proportion of grooming events scored from 24 hr videos, graphed against beam crossing frequency for each genotype (*n* = 4 per genotype). Mean values are fitted with a bounded exponential curve. The relatively high fluctuation at higher frequencies is due to low numbers of these events in controls and fmn flies (*e.g.*, there were only two data points at eight and nine beam crossings for *fmn* and *w^1118^*, respectively; one of each was grooming). The *nf1*^P1^ data are from the same flies as graphed in Figure 3E.

We hypothesized that the bursts of activity recorded by the activity monitors reflected grooming, as follows. When a fly stops patrolling near, or in, the IR beam by chance, and begins grooming, the movements would be expected to cause large numbers of beam breaks in a short period of time. To test this, we placed *nf1*^P1^ flies in activity monitors, and video recorded them for 24 hr while monitoring IR beam breaks in 10 s bins (Figure 3, Supplemental Material, File S1, and File S2). Each event recorded by the activity monitor was located in the time-matched video frames, and marked as either grooming or locomotion (Figure 3, A–D). A single beam crossing event in a 10 s bin represented locomotion 74.6% of the time, and this percentage quickly dropped as the number of beam crossings increased (Figure 3E). All instances of 6+ beam crossings in a 10 s bin were grooming, demonstrating that the high-frequency activity spikes recorded in *nf1*^P1^ mutants represent grooming events rather than locomotion. To confirm that this was the case for similar (though less frequent) activity spikes in wild-type controls, we analyzed videos of *wCS10* flies in the activity monitors. Similar to *nf1*^P1^ mutants, high-amplitude spikes in activity represented mainly grooming events in *wCS10* controls, with all events of 10+ beam crossings per 10 s bin representing grooming (Figure 4F).

Since both activity and grooming were detected by IR activity monitors, previously characterized mutants could have alterations in either or both. This raised the question of how the Nf1 phenotype relates to that of other activity mutants with no known grooming phenotype. One of the most well-characterized activity mutants is the dopamine transporter (DAT) mutant, *fumin* (*fmn*) (Kume *et al.* 2005). To compare the activity profiles of *fmn* and *nf1* mutants, we recorded activity of the mutants and their respective genetic controls, both in IR activity monitors (Figure 4, A–E), and in IR activity monitors with video recording (Figure 4F). *fmn* mutants exhibited an increase in baseline activity, with little of the high-frequency spiking activity that characterized the *nf1* mutants (Figure 4, A–C). There was neither a shift in histograms (Figure 4D), nor a significant difference in mean frequencies, between *fmn* and control flies (Figure 4E). A second cohort of *nf1*^P1^ mutants and *wCS10* flies that were run simultaneously with the fmn flies (Figure 4, D and E) exhibited similar results to the previous experiment (Figure 2, D and E).

**Figure 5:**
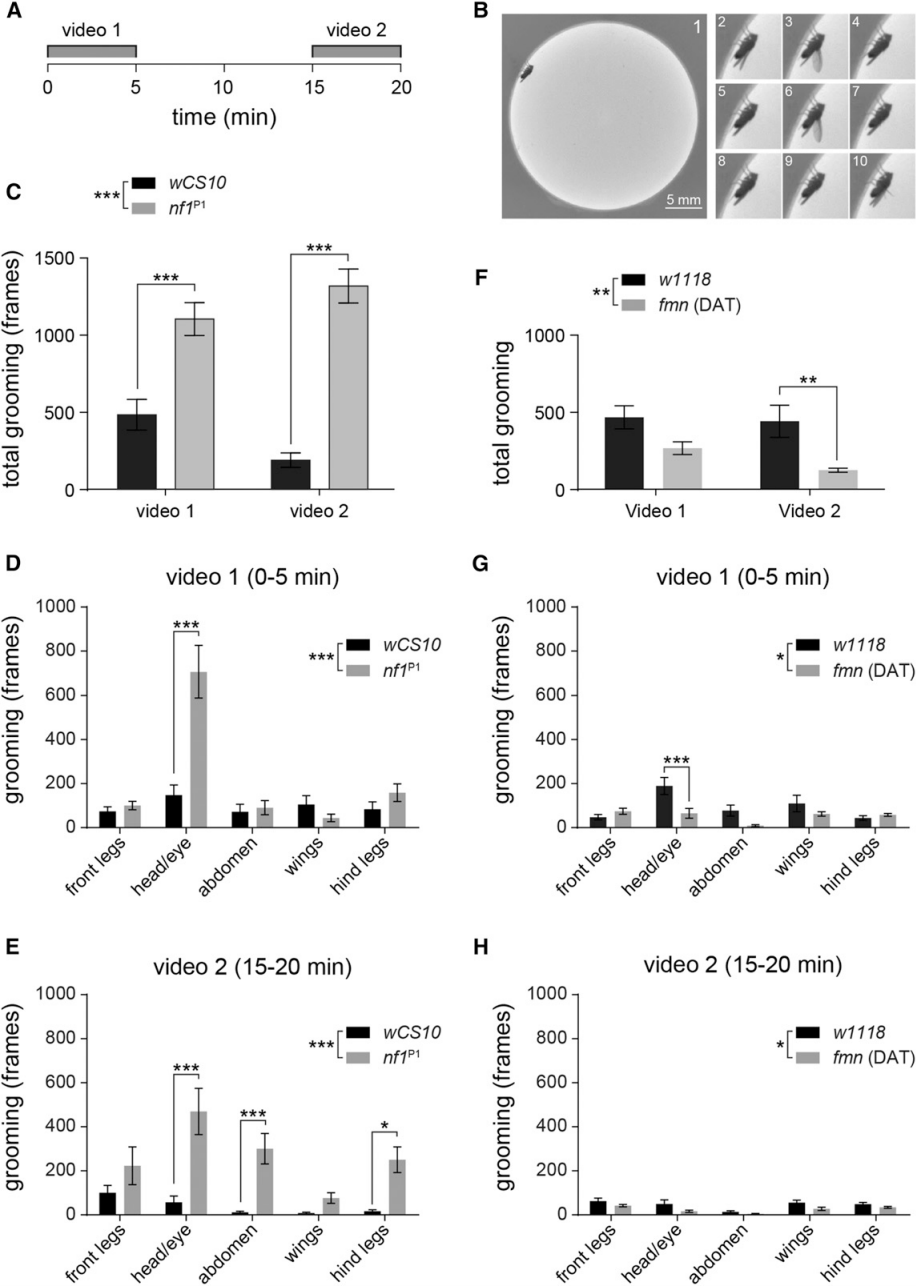
Grooming in *nf1*^P1^ and *fmn* mutants in an open field arena. *nf1*^P1^ mutants exhibited increased grooming that was not seen in *fmn* mutants. Videos were taken at 7.5 frames per sec, and durations are reported in frames. For all panels, the omnibus two-factor ANOVA test result is shown in the legend, and *post hoc* Sidak’s multiple comparisons are shown between individual bars. * *P* < 0.05, ** *P* < 0.01, ** *P* < 0.01; *n* = 18 per genotype. (A) Time periods of video recordings. Flies were loaded at time = 0. (B) Individual, sequential frames of a fly exhibiting wing grooming. Frame 1 is cropped slightly from the original video. Frames 2–10 are cropped in the exact same coordinates. (C) Total grooming time of *nf1*^P1^ mutants. The same flies are further analyzed in (D) and (E). (D) Grooming time for each body region of *nf1*^P1^ mutants for the first 5 min in the open field arena. (E) Grooming time, 15–20 min after transfer to the arena. (F) Total grooming time of *fmn* dopamine transporter mutants. The same flies are further analyzed in (G) and (H). (G) Grooming time for each body region of *fmn* mutants for the first 5 min in the open field arena. (H) Grooming time, 15–20 min after transfer to the arena.

**Figure 6:**
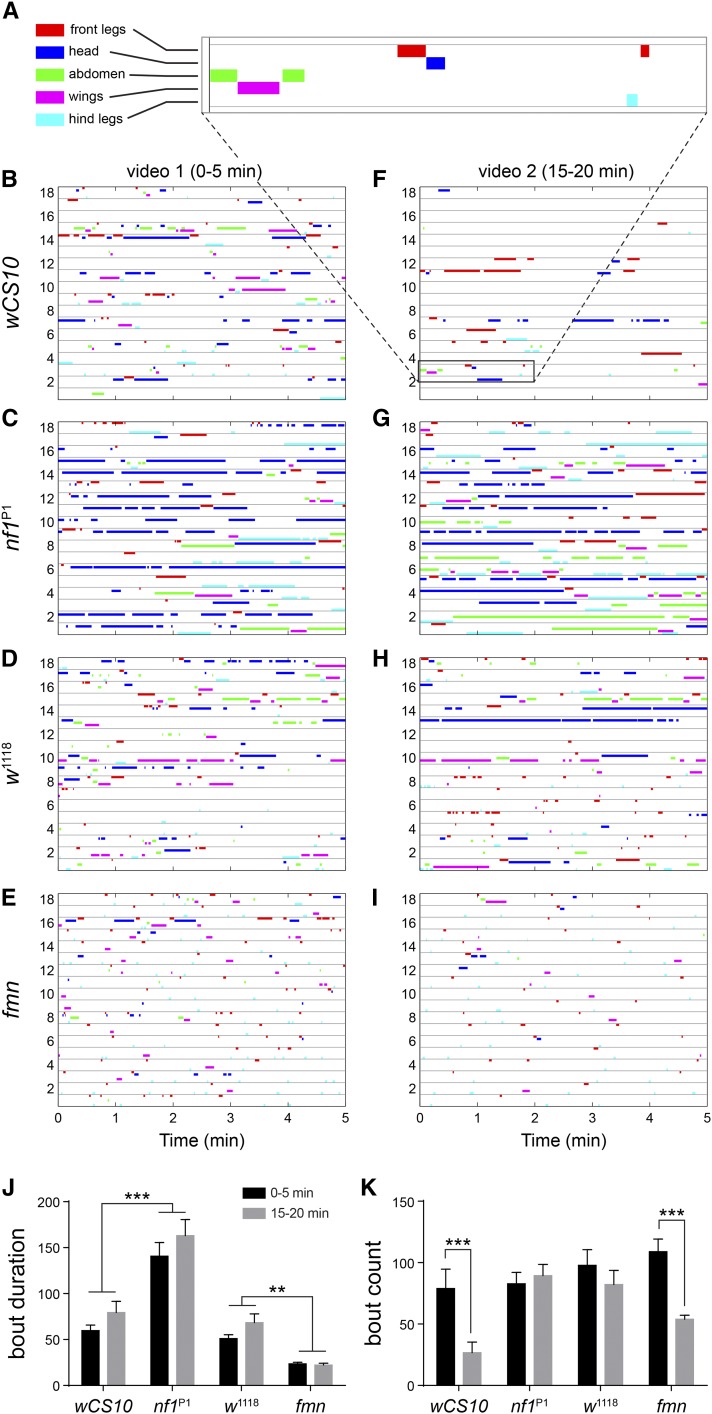
Ethograms of *nf1*^P1^ and *fmn* mutants. (A) Expanded view of a 2 min time segment, with the legend showing color coding for grooming behaviors. (B)–(I) Ethograms of flies of the following genotypes: *wCS10*, *nf1*^P1^, *w^1118^*, *fmn*. Two points are shown: 0–5 min, and 15–20 min. Data are from the same flies as in Figure 5. (J) Duration of individual grooming bouts, in frames, for each genotype and time point. Bars and whiskers represent mean ± SEM. ** *P* < 0.01, *** *P* < 0.001 (ANOVA/Sidak). (K) Number of grooming bouts for each genotype and time point. *** *P* < 0.001 (ANOVA/Sidak). There was no significant difference across genotypes for the 0–5 min videos.

To more completely characterize the grooming phenotypes of *nf1* mutants, we analyzed videos of flies in a backlit open field arena in 5 min intervals (Figure 5, Figure 6, File S3, and File S4). This configuration allowed us to quantify behavior in a less spatially confined environment, providing ample opportunity for locomotion and grooming. Similar to a previous report, we noted that the flies exhibited a tendency toward exploration of the boundary region (Soibam *et al.* 2013). Individual grooming behaviors were scored separately: head (including eyes and/or antennae), front legs, back legs, wings, or abdomen. Since the arena represented a novel environment, we reasoned that the flies may habituate over time. Therefore, videos were collected of each fly at two time points following introduction to the chamber: one immediately after transfer (0–5 min), and a second time after a period of acclimation (15–20 min). When the flies were initially transferred to the open field, *wCS10* controls groomed 21.4% of the time (482.4 ± 99.2 frames out of 2250) (Figure 5C). The *nf1*^P1^ mutants groomed 49.0% of the time (1102.0 ± 106.0 frames), a significant increase over the control (*P* < 0.001; ANOVA/Sidak). After a 15 min acclimation period, the *nf1* mutants showed no decay in activity, and again groomed significantly more than the *wCS10* controls (*P* < 0.001). The magnitude of this effect was striking, with the *nf1* mutants grooming 7× more than controls. Control *wCS10* flies exhibited a trend toward less grooming after the acclimation period, similar to a locomotor decay effect observed in previous studies (Connolly 1967; Liu *et al.* 2007). Although the difference in total grooming time did not reach significance (Figure 5C), there was a significant drop in grooming frequency after the 15 min acclimation period that did not appear in *nf1*^P1^ mutants (Figure 6K). Overall, these data demonstrated that Nf1 loss of function increases grooming frequency. In terms of specific grooming behaviors, in the first video, *nf1*^P1^ flies exhibited significant increases specifically in head grooming (Figure 5D and Figure 6). However, at the time of the second video, elevated grooming was observed across the head, abdomen, and hind legs (Figure 5E and Figure 6, F and G).

Finally, we compared the *nf1* grooming phenotype with *fmn* mutants by analyzing video of *fmn* mutants and *w1118* controls in the open field arena. In contrast to the *nf1* mutants, *fmn* flies exhibited a modest decrease in grooming (Figure 5, F–H). The decrease in total grooming was significant in omnibus tests in both videos (*P* < 0.05; ANOVA), and there was a significant decrease in head grooming in video 1 in File S1 (*P* < 0.001; Sidak). Analysis of bout duration and frequency showed that genotype significantly affected the bout duration, with *nf1*^P1^ flies exhibiting longer bouts, and *fmn* shorter than controls at both time points (*P* < 0.01; ANOVA/Sidak; Figure 6). There was no difference in bout frequency immediately following introduction to the open field (Figure 6K), but, by 15–20 min, the *wCS10* and *fmn* flies had a significantly reduced bout count (*P* < 0.001; ANOVA/Sidak). These data suggest that both Nf1 and DAT loss of function increased total activity levels registered in infrared activity monitors, but that the phenotypes were qualitatively and quantitatively distinct when analyzed in terms of grooming *vs.* locomotion.

## Discussion

The present data demonstrate that loss of Nf1 produces an excessive grooming phenotype in *Drosophila*. The magnitude of the effect is the largest reported to date, to our knowledge, with a sevenfold increase in grooming in an open field arena after 15 min of acclimation. High-resolution analysis and video tracking of *nf1* and *fmn* (DAT) mutants revealed that changes in activity observed in traditional infrared activity monitors can result from changes in locomotor activity and/or grooming. Nf1 flies exhibiting increased grooming showed a histogram skewed toward higher values, with a concomitant increase in mean frequency. In contrast, *fmn* mutants that exhibited increased locomotor activity showed elevated baseline activity that was concentrated on low frequency values, with no significant change in the mean frequency. In other words, they crossed the beam at least once in a larger number of bins, as they patrolled back and forth more consistently, but did not exhibit an increase in the mean number of beam crossing per bin. Therefore, IR activity monitors can be used to observe both locomotion and grooming behavior in *Drosophila*. The contributions of these two components can be estimated by examining activity histograms, single-fly activity traces, and mean frequency of beam-crossing events.

IR activity monitors are commonly used, and we suggest they may be used as a high-throughput screening tool to identify genes or neuronal subsets involved in this complex behavior. This approach complements methods that offer more detail but require more time or specialized resources, such as manual scoring, imaging of dust cleaning (Phillis *et al.* 1993; Seeds *et al.* 2014), and machine learning algorithms (Kain *et al.* 2013; Berman *et al.* 2014). Multiple activity phenotypes could result in changes in beam crossing frequency per unit time, including seizures or locomotor deficits. Video analysis is necessary to confirm the nature of putative grooming phenotypes identified using the above criteria. We note that sampling of grooming in the activity events in the monitors is sparse, since flies must groom in a precise location to break the IR beam at high frequency. Given this consideration, ample numbers of flies and time should be used to ensure that enough grooming events are sampled. In this study, we measured 32 flies per genotype, for a minimum of 5 d, and were able to reliably detect grooming increases in Nf1 deficient flies.

A previous study reported that knock-down of Nf1 and DAT produce similar nighttime hyperactivity patterns using IR activity monitors, which was interpreted as a shared locomotor signature (van der Voet *et al.* 2015). Genomic mutations in Nf1 and DAT (*fmn*) mutants produced distinct phenotypes in our hands, both in terms of diurnal activity patterns, and grooming. The diurnal activity pattern observed in *fmn* mutants is consistent with their previously-reported L:D phenotype (Kume *et al.* 2005). Our data suggest that loss of either Nf1 or DAT increased activity as measured by IR beam breaks. However, these activity phenotypes were distinct, resulting from increased grooming and locomotion, respectively. Our data do not rule out the possibility that an increase in locomotion in *nf1* mutants could be present along with the excess grooming phenotype, as measurements from IR activity monitors represent a composite of activity and grooming. Overall, these results highlight that analysis of IR beam breaks must be supplemented by histogram and video analysis in order to attribute changes in activity to locomotion and/or grooming.

Grooming has been reported to follow the pattern of a suppression hierarchy (Seeds *et al.* 2014). In this model, grooming of one body part suppresses grooming of other body parts, allowing the animal to complete these mutually exclusive motor tasks in an orderly sequence. When flies are covered in dust, the grooming sequence is eyes > antennae > abdomen > wings (Seeds *et al.* 2014). In the present study, flies lacking Nf1 exhibited increased grooming. When flies were transferred to a new environment, they initially groomed only their head. After 15 min, they groomed the head + abdomen + legs excessively. This may indicate that loss of Nf1 affects neurons either at the top of the grooming hierarchy (head grooming circuits), or in neurons that control grooming drive (*e.g.*, sensory neurons), rather than a circuit specific to a body part lower in the suppression hierarchy. Under these assumptions, introduction to a new environment triggers an overall increase in grooming drive, stimulating grooming down the suppression hierarchy in a temporally sequential manner. The fly starts grooming from the head and later incorporates grooming of other body parts. It is notable that sensory neurons from Nf1 +/− mice are hyperexcitable (Wang *et al.* 2005), though behavioral effects of this sensitivity are unclear (O’Brien *et al.* 2013). If sensory neurons in flies are also hypersensitive, handling them, and transfer to a new environment, could theoretically trigger increased grooming. Alternatively, the enhanced grooming we observe could result from a central disinhibition of grooming circuits. Nf1 +/− mice have dysregulated GABAergic signaling in the amygdala (Molosh *et al.* 2014), hippocampus (Costa *et al.* 2002; Cui *et al.* 2008), and cortex (Shilyansky *et al.* 2010). The *Drosophila* model is a tractable system in which to investigate the contributions of both sensory and central circuits to Nf1 behavioral phenotypes.

Known signaling functions of Nf1 are highly conserved, and the loss of Nf1 presumably affects neuronal function in fundamentally similar ways across taxa. The most well-characterized biochemical function of Nf1 is negative regulation of Ras signaling via its GAP-related domain (Cichowski and Jacks 2001; Costa *et al.* 2002). However, Nf1 is a large, 320 kDa protein, and most of its domains have unknown function. Several lines of evidence suggest that Nf1 could have pleiotropic signaling roles. In addition to Ras hyperactivation, Nf1 deficiency affects cAMP levels in multiple cell types (possibly indirectly) (The *et al.* 1997; Tong *et al.* 2002; Dasgupta *et al.* 2003; Hannan *et al.* 2006; Diggs-Andrews *et al.* 2013; Walker *et al.* 2013; Wolman *et al.* 2014). Domain-specific rescue suggested that two different domains of the Nf1 protein may independently influence distinct forms of memory (Ho *et al.* 2007). In addition, Nf1 binds multiple other proteins, including tubulin and 14-3-3 proteins, and may regulate multiple signaling cascades, possibly in a cell-type-specific manner (Ratner and Miller 2015). Uncovering genetic modifiers of NF-1-related cellular dysfunction would provide potential new targets for treating this disorder. Human NF-1 phenotypes exhibit variable penetrance, yet have both high concordance between monozygotic twins, and poor genotype–phenotype correlation (Ratner and Miller 2015). This suggests that unknown genetic modifiers exert strong influence over the course of the disease (Easton *et al.* 1993; Rieley *et al.* 2011; Pemov *et al.* 2014). *Drosophila* are an excellent model organism to study signaling/signal transduction, genetic interactions and modifiers, and fundamental cellular physiology. The large Nf1 phenotype provides a potentially powerful platform to dissect the alterations in signaling cascades, and ultimately neuronal function, that result from neurofibromatosis-1.

## 

## Supplementary Material

Supplemental Material
