# Supplementary material for: Neurofibromin Loss of Function Drives Excessive Grooming in Drosophila
Source: G3 (Bethesda). 2016 Feb 18;6(4):1083–93. doi: 10.1534/g3.115.026484 (PMC4825643; doi:10.1534/g3.115.026484)
Supplement: Supplemental Material [file supp_6_4_1083__index.html]

Neurofibromin Loss of Function Drives Excessive Grooming in Drosophila — Supplemental Material 

# Neurofibromin Loss of Function Drives Excessive Grooming in *Drosophila*

## Supplemental Material for King *et al.*, 2016

**Files in this Data Supplement:**

- File S1 - Video of an *nf1*P1 fly grooming in the infrared beam, performing primarily wing grooming. This grooming epoch is the same that is graphed in Fig. 3B,C. (.mp4, 877 KB)
- File S2 - Video of an *nf1*P1 fly walking past the infrared beam. This is the same fly in File S1 and the locomotion event graphed in Fig. 3B,D. (.mp4, 146 KB)
- File S3 - A *wCS10* fly grooming in the open field arena. This video is from fly 3 in Fig. 6F, and corresponds to the 2-min period expanded in Fig. 6A. This time segment was selected to highlight the full grooming repertoire. (.mp4, 344 KB)
- File S4 - Representative video of an *nf1*P1 fly grooming in the open field arena. This video is from the 1-3 min time period from fly 3 in Fig. 6G. (.mp4, 232 KB)
